# A Pilot retrospective analysis of alpha-blockers on recurrence in men with localised prostate cancer treated with radiotherapy

**DOI:** 10.1038/s41598-020-65238-z

**Published:** 2020-05-18

**Authors:** Jordan Hart, Briohny Spencer, Catherine M McDermott, Russ Chess-Williams, Donna Sellers, David Christie, Shailendra Anoopkumar-Dukie

**Affiliations:** 1 Menzies Health Institute, Griffith University, Queensland, Australia; 2 School of Pharmacy and Pharmacology, Griffith University, Queensland, Australia; 3 Centre for Urology Research, Bond University, Gold Coast, Queensland Australia; 4 Genesis Cancer Care, Gold Coast, Queensland Australia; 5 Quality Use of Medicines Network, Griffith University, Queensland, Australia

**Keywords:** Cancer, Prostate

## Abstract

While alpha-blockers are commonly used to reduce lower urinary tract symptoms in prostate cancer patients receiving radiotherapy, their impact on response to radiotherapy remains unknown. Therefore, this pilot study aimed to retrospectively determine if alpha-blockers use, influenced response to radiotherapy for localised prostate cancer. In total, 303 prostate cancer patients were included, consisting of 84 control (alpha-blocker naïve), 72 tamsulosin and 147 prazosin patients. The main outcomes measured were relapse rates (%), time to biochemical relapse (months) and PSA velocity (ng/mL/year). Recurrence free survival was calculated using Kaplan-Meier analysis. Prazosin significantly reduced biochemical relapse at both two and five-years (2.72%, 8.84%) compared to control (22.61%, 34.52%). Recurrence free survival was also significantly higher in the prazosin group. This remained after multivariable analysis (HR: 0.09, 95% CI: 0.04–0.26, p < 0.001). Patients receiving prazosin had a 3.9 times lower relative risk of biochemical relapse compared to control. Although not statistically significant, tamsulosin and prazosin extended recurrence free survival by 13.15 and 9.21 months respectively. We show for the first time that prazosin may reduce risk of prostate cancer recurrence and delay time to biochemical relapse and provides justification for prospective studies to examine its potential as an adjunct treatment option for localised prostate cancer.

## Introduction

Prostate cancer is the fourth most prevalent cancer worldwide and the most common solid tumour among males. In Australia, an estimated 17,729 new cases of prostate cancer are diagnosed each year and it is the second leading cause of cancer related death among Australian men^1^. Approximately 80% of new diagnoses are clinically localised^2^, with a five-year survival rate of approximately 95%^1^. However, the cancer will eventually transition to castrate resistant disease. Following this transition the disease is ultimately incurable^3^ with a median survival of 14 months^4^.

Radiotherapy is an important treatment modality being used in approximately 60% of localised prostate cancer patients^5^. While it is an effective treatment with a five-year survival of 98.8%^6^, it is limited by the associated adverse effects. The toxicities associated with radiotherapy can be both acute and chronic in nature, and mainly impact the lower urinary tract causing complications which include radiation cystitis and bladder outlet obstruction, which can significantly affect a patient’s quality of life^7–9^. α_1_-adrenoceptor antagonists such as tamsulosin and prazosin are commonly used to relieve the voiding symptoms associated with benign prostatic hyperplasia. In addition, these drugs are recommended either prophylactically^10^ or at onset of lower urinary tract symptoms in men treated with radiotherapy for prostate cancer^11^. While α_1_-adrenoceptor antagonists are widely used clinically, their impact on the outcomes of radiotherapy have to date not been considered.

Numerous studies suggest that the α_1_-adrenoceptor antagonists, may possess both cytotoxic and antitumour effects in various *in vitro* models of prostate cancer, exerting cytotoxic effects via mechanisms independent to that of the α_1_-adrenoceptor blockade^12–17^. There is substantial evidence that the quinazoline α_1_ antagonists (terazosin, prazosin and doxazosin) display cytotoxicity in prostate cancer cell lines^14,18–20^, effects that are not observed with the sulphonamide derivative tamsulosin^21^. This cytotoxicity has been observed with high concentrations of the α_1_-adrenoceptor antagonists *in vitro* and is thought to occur primarily via apoptotic cell death, however the complete range of cell death mechanisms remains to be fully understood^13,22^. *In vivo* studies in mice have shown that these effects also occur at clinically relevant doses, with reduction in tumour growth, metastasis and decreased angiogenesis observed in models of prostate cancer^23,24^.

By comparison to *in vitro* and *in vivo* studies, the clinical evidence in prostate cancer patients is lacking with studies only evaluating the role of α_1_-adrenoceptor antagonists in reducing prostate cancer incidence rather than as a treatment option. Harris, A. *et al*., conducted an observational cohort study on men treated with either doxazosin or terazosin for benign prostatic hyperplasia or hypertension^25^. The study found that the incidence of prostate cancer in treated men compared to untreated men was lower with 7.6 fewer cases of prostate cancer per 1000 treated men^25^. Similarly, Yamada *et al*., found that the incidence of prostate cancer was significantly lower for patients treated with naftopidil when compared to tamsulosin^26^. The only study to investigate the cytotoxicity of these agents in humans was performed by Keledjian *et al*., in benign prostatic hyperplasia patients^27^. These authors reported that terazosin increased the apoptotic index and reduced prostate vascularity in these patients^27^.

To our knowledge, there have been no studies to date that have investigated these agents for their potential to affect outcomes for radiotherapy for localized prostate cancer. Therefore, the aim of this study was to determine the potential of the α_1_-adrenoceptor antagonists as a possible adjunct treatment in patients receiving radiotherapy for localised prostate cancer. This was achieved retrospectively at Genesis Cancer Care Southport and investigated the ability of these agents to delay time to biochemical relapse and reduce prostate cancer recurrence.

## Materials and Methods

### Patients and ethics

Data from patients with histologically proven adenocarcinoma of the prostate who had received radiotherapy at Genesis Cancer Care Southport (2000 and 2017) was retrospectively analysed. Human research ethics was approved by Uniting Care Health Queensland for data collection at their facility (Reference number: 2014.20.126) and approval was also gained from the Griffith University ethics committee (Reference number: PHM/10/14/HREC). A waiver of informed consent was also granted by Uniting Care Health Queensland. Patient groups included (1) control who were α_1_-adrenoceptor antagonist naïve, (2) men receiving tamsulosin and (3) prazosin at the time of radiotherapy.

### Inclusion and exclusion criteria

The Genesis Cancer Care database contained records for over 10,000 patients, which was first refined using a number of specific keywords: tamsulosin, Flomaxtra, prazosin and Minipress. A search including these keywords was used to identify patients who had received an α_1_-adrenoceptor antagonist (tamsulosin and prazosin treatment groups), while a search excluding these terms identified the control group that had not received an α_1_-adrenoceptor antagonist. Only prostate cancer patients who had received radiotherapy as part of their treatment plan were included in the study. Additional criteria for inclusion were, age > 45 years old and no pre-existing medical conditions or other treatments that may have affected PSA. The exclusion criteria were: age < 45 years old, patients with any other cancer or metastatic disease, patients who had never received radiotherapy and patients with any pre-existing medical conditions or treatments that may have affected PSA. Patients with insufficient medical data were also excluded for comprehensiveness.

### Data collection and end points

Following the identification of eligible patients, data was systematically collected using the CAS8 medical records database. The database contained notes by the radiation oncologist, referral letters, correspondence with other health professionals, pathology and relevant medical imaging results. Baseline demographic characteristics including age at diagnosis, Gleason score, tumour staging, and any treatment modalities were collected. PSA values were recorded periodically from diagnosis up to 120 months (10 years) if available. The main outcomes measured were percentage relapse, time to biochemical relapse and PSA velocity. Although doses were not available for each patient, typical dose ranges used at the clinic for the patients were 1–2 mg/day for prazosin and 0.4 mg tamsulosin/day. Treatments were started during radiotherapy and then continued for 2–3 months post treatment until LUTS resolved.

### Risk stratification

Risk groups established by the National Comprehensive Cancer Network (NCCN) were used to stratify patients according to PSA at diagnosis, Gleason score and tumour staging. Low risk patients (85–94% five-year survival) were defined as those with a diagnostic PSA of < 10 ng/mL, Gleason score of ≤ 6 and tumour staging T1c or T2a. Intermediate risk patients (68–84% five year survival) were defined as those with a diagnostic PSA > 10 to 20 ng/mL or a Gleason score of 7 or tumour stage T2b. High risk patients (43–67% five year survival) were defined as those with a diagnostic PSA > 20 ng/mL or a Gleason score of 8–10 or tumour staging T2c/T3.

### Statistical analyses

Numerical data was expressed as mean ± standard deviation and median. GraphPad Prism (version 7) and IBM SPSS Statistics 26 software was used for statistical testing. A two by two contingency table with Fisher’s exact test was used to compare the proportion of men who relapsed between groups. Analysis of variance (ANOVA) with Tukey-Kramer post hoc testing was used to compare time to biochemical relapse and PSA velocity. P values < 0.05 were considered significant. Recurrence free survival (%) was calculated and analysed using the Kaplan Meier method and log rank test (Mantel-Cox) on GraphPad Prism (version 7) to test for significance. Cox regression analysis was performed using IBM SPSS Statistics 26 software and was used to compare survival of patients in the tamsulosin and prazosin group to survival of those in the control group. Age, stage at diagnosis, Gleason score and initial PSA were used as covariates in the regression model.

## Results

### Patient demographics

In total, 303 prostate cancer patients with localised disease at Genesis Cancer Care were identified for inclusion in the study. This consisted of 84 control patients, 72 tamsulosin treated patients and 147 prazosin treated patients. Based on the exclusion criteria described in the methods section, 219 other patients were excluded from the study.

The mean age at diagnosis for the whole cohort was 68.38 ± 6.71 years. In the control group the mean age was 68.54 ± 7.27 years while in the tamsulosin and prazosin groups the mean age was 66.81 ± 5.52 and 69.06 ± 5.68 years respectively (Table 1). A statistical comparison of age between groups yielded only a minor significant difference between the tamsulosin and prazosin groups (*p = 0.0210).

**Table 1:** Demographics of prostate cancer patients at diagnosis.

**Characteristic at diagnosis**	**Control (n** = **84)**	**Tamsulosin (n** = **72)**	**Prazosin (n** = **147)**
**Age (years)**			
Mean ± SD	68.54 ± 7.27	66.81 ± 5.52	69.06 ± 5.68*p = *0.021*
Median	69	70	67
**PSA (ng/mL)**			
Mean	16.76 ± 15.89	13.63 ± 10.26	14.97 ± 17.52
Median	12	9.40	11
**Risk Stratification**	Column1	Column2	Column3
Low	8 (9.50%)	6 (8.30%)	6 (4.10%)
Intermediate	41 (48.80%)	21 (29.20%)	57 (38.80%)
High	35 (41.70%)	45 (62.50%)	84 (57.10%
**Tumour staging**			
T1	13 (15.50%)	18 (25.00%)	35 (23.80%)
T2	43 (51.20%)	25 (34.70%)	70 (47.60%)
T3	12 (14.30%	28 (38.90%)	25 (17.00%)
T4	0 (0.00%)	0 (0.00%)	3 (2.10%)
Unknown	16 (19.00%)	1 (1.40%)	14 (9.50%)
**Gleason sum**			
3–6	29 (34.50%)	7 (9.70%)	15 (10.20%)
7	32 (38.10%)	34 (47.20%	64 (43.50%)
8–10	21 (25.00%)	30 (41.70%)	64 (43.50%)
Unknown	2 (2.40%)	1 (1.40%)	4 (2.80%)

(*Prazosin vs tamsulosin).

The mean PSA at diagnosis in the control, tamsulosin and prazosin groups was 16.76 ± 15.89, 13.63 ± 10.26 and 14.97 ± 17.52 ng/mL respectively with no significant difference in PSA at diagnosis identified between groups.

Following stratification into risk groups using the NCCN guidelines described in the methods section, it was observed that a higher proportion of patients in both tamsulosin (62.50%) and prazosin groups (57.10%) were identified as high risk when compared to control (41.70%). Similarly, both treatment groups were shown to have a higher proportion of patients with locally advanced disease (defined by the European Association of Urology (EAU) as tumour staging at diagnosis of T3 or T4)^28^. In the tamsulosin group 38.90% of patients were diagnosed with T3/T4 disease while 19.10% of prazosin patients received the same diagnosis. In comparison, only 14.30% of control patients had T3 disease with no patients identified as having T4 disease at diagnosis. Further to this, prostate biopsy pathology results revealed that 41.50% of tamsulosin patients and 43.50% of prazosin patients had a Gleason score of 8–10 indicative of high grade, poorly differentiated prostate cancer^29^. In contrast, only 25% of control patients had a Gleason score of 8–10 at initial diagnosis. The full patient demographics are shown in Table 1.

### Relapse rates

During the observational period, 78 patients (25.74%) were diagnosed with relapsed prostate cancer. This included 35 control, 28 tamsulosin and 15 prazosin patients. Percentage relapse for the control group was found to be significantly higher at both the two and five-year time points when compared with prazosin (*p < 0.001) (Table 2). Similarly, percentage relapse for tamsulosin was significantly higher than prazosin (*p = 0.011, *p = 0.0019). No significant difference was identified between the control and tamsulosin groups (p = 0.3905, p = 0.2231).

**Table 2:** Two and five-year percentage (%) relapse rates in control, tamsulosin and prazosin treatment groups.

**Relapse rates (%)**	**Control**	**Tamsulosin**	**Prazosin**
% patients relapse - 2 years	22.61% (n = 19)***P < *0.001*	15.27% (n = 11) *p = *0.011*	2.72% (n = 4)
% patients relapse - 5 years	34.52% (n = 29) ***p < *0.001*	25.00% (n = 18) *p = *0.0019*	8.84% (n = 13)

(*vs Prazosin).

The control group had a 34.52% cumulative incidence at the five-year point while the cumulative incidence for prazosin was 8.84%. This resulted in a calculated risk ratio of 0.256 (95%CI: 0.141, 0.465) and risk difference of −0.257. A comparison was also made between the two α1-adrenoceptor antagonist treatment groups which resulted in a risk ratio of 0.354 (95%CI: 0.1837, 0.6813) and risk difference of −0.162.

**Figure 1:**
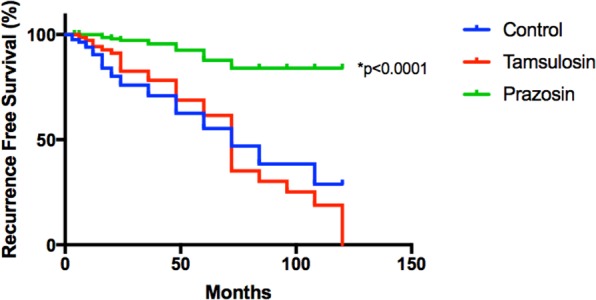
Kaplan Meier plot of recurrence free survival (%) in control, tamsulosin and prazosin treatment groups for a period of 120 months (10 years).

Recurrence free survival is an important measure of treatment effectiveness and has been used to evaluate a number of treatments for localised prostate cancer^30–32^. In this study, Kaplan Meier curves (Fig. 1) were used to compare recurrence free survival (%) between the control and treatment groups. Analysis indicated that recurrence free survival (%) was significantly higher and median survival was extended in the prazosin group when compared to the control and tamsulosin groups (median recurrence free survival undefined in the prazosin group vs 72 months in both control and tamsulosin groups). Median survival is reported as undefined for the prazosin group as survival exceeds 50% at the ten-year mark. Log-rank (Mantel-Cox) testing indicated a significant difference between survival curves (*p < 0.0001). The results for unadjusted COX regression analyses for tamsulosin and prazosin groups compared to control indicate that tamsulosin had no significant effect on survival (HR: 0.63, 95% CI: 0.35–1.14, p = 0.124) when compared to control, however use of prazosin was associated with a significant increase in survival (HR: 0.17, 95% CI: 0.08–0.35, p < 0.001). When adjusted for covariates; age, staging, Gleason score and initial PSA, the same trend was evident, with a stronger significant association between greater survival in prazosin patients with a HR of 0.09 (95% CI: 0.04–0.26, p < 0.001). The adjusted hazard ratio for the tamsulosin group was 0.46 (95% CI: 0.20–1.06, p = 0.067).

### Time to biochemical relapse

For this study, biochemical relapse was defined by the phoenix definition of a PSA rise of >2 ng/ml above the nadir which was identified as the most robust determinant of patient outcomes^33^. This definition allowed for the calculation of time to biochemical relapse in each group as shown in Table 3. The mean time to biochemical relapse in the control group was 35.71 ± 27.36 months compared to 48.86 ± 31.73 and 45 ± 19.00 months in the tamsulosin and prazosin groups. Univariate analyses showed time to biochemical relapse did not differ significantly between groups (p = 0.1258). Despite the lack of statistical significance, tamsulosin and prazosin were found to extend time to biochemical relapse by 13.15 and 9.29 months respectively when compared to the control group. Interestingly, median time to biochemical relapse in both tamsulosin and prazosin groups were equal (48 months) however mean time was increased in the tamsulosin group.

**Table 3:** Time to biochemical relapse (months) in control, tamsulosin and prazosin treatment groups.

Time to biochemical relapse (months)	**Control (n** = **35)**	**Tamsulosin (n** = **28)**	**Prazosin (n** = **15)**
Mean ± SD	35.71 ± 27.36	48.86 ± 31.73	45 ± 19.00
Median	24.00	48.00	48.00
Range	105.00	114.00	68.00

Data is reported as mean ± standard deviation, median and range.

### PSA velocity

PSA velocity is used as a prognostic factor to determine risk of relapse after treatment^34^. This was calculated using linear regression analysis^31,35,36^ and all available PSA values from three months’ post radiotherapy up until the last available pathology result were included (Table 4). For relapsed patients, only PSA values prior to biochemical relapse were included in this calculation. As shown in Table 4, prazosin patients had a lower PSA velocity (0.31 ng/mL/year) when compared to the control (2.98 ng/mL/year) and tamsulosin (3.36 ng/mL/year) groups.

**Table 4:** Comparison of PSA velocity (ng/mL/year) for all patients in the control, tamsulosin and prazosin groups.

**PSA velocity (ng/mL/year)**	**Control**	**Tamsulosin**	**Prazosin**
Mean ± SD	2.98 ± 10.99	3.36 ± 15.80	0.31 ± 2.15
Median	0.075	0.086	0.010

Data is reported as mean ± standard deviation and median.

## Discussion

Prostate cancer is extremely treatable with a five-year survival rate of 95%, however it is estimated that between 20–50% of patients will experience biochemical relapse within 10 years depending on treatment choice^37^. Currently, only a limited number of relatively invasive treatment options exist such as radiotherapy, radical prostatectomy, androgen deprivation and chemotherapy however these are associated with a number of adverse effects, toxicities as well as the development of treatment resistance. Therefore, there is an urgent need to identify effective primary or adjunct treatment options and the α_1_-adrenoceptor antagonists may represent one of these options. Numerous experimental studies have demonstrated the cytotoxic activity of the quinazoline based α_1_- adrenoceptor antagonists in both androgen dependent and castrate resistant cell lines^12–20,38,39^. This cytotoxic effect was not demonstrated with the sulphonamide derivative tamsulosin. The cytotoxic effects of the quinazoline α_1_- adrenoceptor antagonists appears to be mediated by apoptotic and antiangiogenic effects and is believed to occur via a mechanism largely independent to that of the α_1_-adrenoceptor blockade^39^. In addition, *in vivo* studies have shown these agents are able to significantly reduce tumour weight and suppress metastasis in mice^40^. Although sufficient *in vitro/in vivo* evidence exists, the limited human evidence has primarily evaluated the potential of the α_1_-adrenoceptor antagonists in reducing the risk of prostate cancer. To our knowledge, there are no studies to date which have investigated the role of the quinazoline α_1_-adrenoceptor antagonists as a novel adjunct treatment option for prostate cancer and as such this is the first study to retrospectively investigate the ability of these agents in delaying time to biochemical relapse and reducing recurrence in prostate cancer patients. In our study, 303 patients were identified for inclusion. All patients had histologically proven adenocarcinoma of the prostate and all patients had received some form of radiotherapy as part of their treatment regimen.

Three primary outcomes were used to assess the effectiveness of prazosin and tamsulosin in delaying or preventing prostate cancer biochemical relapse. These outcomes included relapse rate (%), time to biochemical relapse (months) and PSA velocity (ng/mL/year). Relapse rates and time to biochemical relapse are outcomes which are frequently used in investigating the effectiveness of prostate cancer treatments^41,42^. Here we show that prazosin, at clinically used doses, significantly reduced the number of patients who experienced biochemical relapse at both the two and five-year points when compared to the control and tamsulosin groups. Kaplan Meier survival analysis demonstrated that recurrence free survival (%) was strikingly higher in the prazosin group and median survival was significantly extended. These results provide strong evidence that the quinazoline α_1_-adrenoceptor antagonist prazosin can improve treatment outcomes by dramatically reducing the number of patients who experience biochemical relapse following radiotherapy. In contrast, the sulphonamide derivative tamsulosin was found to have no significant effect on both two and five-year relapse rates or recurrence free survival.

Evaluation of risk ratio using the five-year percentage relapse rates indicated that prazosin patients have a 3.9 times lower relative risk of biochemical relapse when compared to control. Interpretation of the risk difference indicated that 257 fewer prostate cancer cases would have developed per 1000 treated men, in this case 38 additional cases of cancer recurrence could have been expected had the patients not received prazosin. This is consistent with previously reported observations which found benign prostatic hyperplasia patients treated with a quinazoline α_1_- adrenoceptor antagonist resulted in a significantly reduced number of prostate cancer cases when compared to control patients (7.6 per 1000)^25^, however provides conflicting evidence to the study performed by Murtola *et al*., which indicated that α_1_-adrenoceptor antagonist use resulted in increased risk of biochemical relapse in prostate cancer^43^.

Time to biochemical relapse was defined by the phoenix definition of a PSA rise of > 2 ng/ml above the nadir and this outcome was compared between the control and two treatment groups^44^. Interestingly, although the two and five-year percentage relapse were significantly lower in the prazosin group when compared with the control and tamsulosin groups there was no significant difference in the time to biochemical relapse. Despite the lack of significance, time to relapse in both the tamsulosin and prazosin groups was extended by 13.15 and 9.21 months respectively. In the setting of cancer treatment with the objective of prolonging life, this is a clinically significant finding.

While methods such as PSA monitoring and PSA doubling time have traditionally been used as a prognostic tool for prostate cancer, PSA velocity is now being increasingly used due to its potential for increased specificity in prostate cancer detection^45,46^. Our results indicated that prazosin patients had a much lower PSA velocity when compared to the control and tamsulosin groups. While no statistical significance was identified between the control and prazosin group, this result is of clinical significance as previous studies have shown that a PSA velocity above 0.35–2 ng/mL/year can increase the risk of prostate cancer between 5.3 and 10 fold^46,47^. The prazosin group was the only cohort to remain below this threshold in this study indicating that this agent plays a key role in reducing the risk of recurrence in prostate cancer patients. Consistent with *in vitro* evidence, tamsulosin demonstrated no significant effect on lowering PSA velocity and therefore reducing the risk of prostate cancer.

Consistent with *in vitro* and animal studies demonstrating the cytotoxic actions of the quinazoline α_1_-adrenoceptor antagonist prazosin, we show here for the first time that prazosin, at clinically relevant doses is able to significantly reduce the risk of prostate cancer recurrence as well as delay time to biochemical relapse in prostate cancer patients following radiotherapy. Although the sulphonamide derivative tamsulosin delayed time to biochemical relapse, there was no significant effect on two and five-year relapse rates or PSA velocity. This is consistent with *in vitro* evidence which demonstrated no cytotoxic actions of tamsulosin even at suprapharmacological concentrations^21^. Therefore, while some of the actions of prazosin may involve α_1_ adrenoceptor blockade it is likely that the majority of the effects observed in this study are independent to the α_1_-adrenoceptor as seen in *in vitro* and animal studies^13^. While the effects may involve several of the *in vitro* targets shown to mediate cytotoxicity such as VEGF, EGFR, HER2/Neu, caspase 8/3, topoisomerase 1 and other mitochondrial apoptotic inducing factors, further studies investigating the mechanisms are needed^13,23,48,49^. However, despite a lack of clear mechanism/s, the results from this study provide a strong argument for the clinical use of the quinazoline α_1_- adrenoceptor antagonists in the treatment of prostate cancer. Despite this, further investigation from a prospective clinical trial using these agents must be performed to establish their role as either an adjunct or primary treatment option.
